# The Kennedy Matter

**Published:** 1892-08

**Authors:** 


### Correspondence.

### "THE KENNEDY MATTER."

### Dr. James Kennedy Speaks.—"One Tale Holds Good till Another is Told."

In the July number of the *Texas Health Journal* is published an open letter to Prof. H. A. West, Secretary of the Texas State Medical Association. The document is declared to be an official communication from the West Texas Medical Association, and bears the signatures of the President and Secretary. The seal of the West Texas Medical Association is likewise attached to it. In this letter they accuse Dr. West of "having attempted to forestall the opinion and prejudice the judgment of the Judicial Council" in my favor. They accuse the Judicial Council of unfairness and illegality of action. They express their defiance of the authority of the Texas State Medical Association by refusing to correct the irregularities and non-conformities to their constitution and by-laws (of which they have been proven guilty), as they are advised to do in the report of the Judicial Council. And lastly, they make the very grave charge against Dr. West of having abstracted a portion of the record of my trial before the Judicial Council.

In the face of the publication of this official (?) document, I feel it incumbent upon me to set forth the main *facts* in relation to my expulsion from this autocratic body (W. T. M. A.) and my trial before the State Medical Association, and in this way illustrate how much truth, justice and loyalty is expressed in the "official open letter" which they thought proper to have published in that great exponent of ethics (?) known as the "*Texas Health Journal.*"

I joined the West Texas Medical Association in October, 1890, and was immediately thereafter elected to the office of secretary and treasurer. I continued in office until May, 1891, when, owing to certain discourtesies, not to say affronts, tendered me by certain members present at a meeting of the Association during the discussion of the question "The Best Method of Resuscitating the Drowned," which was being discussed, I resigned.

The position taken by the old "war horses" of the Association was that no water entered the lungs at all, and, consequently, the practice of placing the individual across a barrel and rolling him, was a senseless procedure. My opinion was asked for, and when I delivered a few remarks showing that water did enter the lungs, and in abundance, and that the reason why water entered the lungs was because there being no obstruction to its exit, the air escaped, being displaced by the water. After many discourteous remarks, the president adjourned the meeting while I still had the privilege of the floor.

I was succeeded in office by Dr. Russell Caffery, to whom I presented my resignation. Some months after this, Dr. Caffery became disgusted with the management of the Association and tendered his resignation.

About this time the "faithful few" who remained formed a mutual admiration society. They numbered a quorum (seven), and with this uumber they held an annual election of officers. Then the campaign opened—they were determined to crush out all who would not bow to their despotic rule. I was the first man assailed. The sin of which I was charged was that of allowing my name to appear in the daily prints. A sample of my sin was clipped from a daily paper. The paper stated that "Dr. Kennedy, assisted by Dr. Caffery, had performed an operation on Miss ———, that the patient was resting easy, and that hopes of her speedy recovery were entertained."

The next day brought forth the following "courteous" letter spoken of in the "open letter" sent to Prof. West by the West Texas Medical Association: (?)

SAN ANTONIO, TEXAS, Jan. 27th, 1892.

*Dr. James Kennedy:*

DEAR SIR: Your attention is hereby invited to the purport of an article which appeared in the morning paper of the 26th inst., relative to a certain case and operation attended by yourself and Dr. Caffery. Such *advertisement* is a *flagrant violation* of Sec. 3, Art. 1, of the duties of physicians to each other and to the profession at large; of the code of ethics of the American Medical Association, and it becomes our duty to *demand of you an explanation* of the appearance of the same.

C. E. R. KING, M. D.,
J. J. BLAND, M. D.,
E. F. HERTZBERG, M. D.,
Board of Censors.

The "grossly insulting" reply alluded to in the official (?) communication reads as follows:

*To the Board of Censors of the West Texas Medical Association:*

DEAR SIRS: Your favor of Jan. 27th to hand, and in reply will say that as an expression of the supreme contempt in which I hold your meddlesome and unnecessary interference with my affairs, and my utter indifference as to the question asked, I at first concluded to take no notice of your communication whatever. But on second thought it occurred to me as a remote possibility that you might be laboring under a misapprehension; that you might not know that my resignation as a member of your honorable body was tendered last October (three months previous to receiving the above letter), and that since that time I have not considered myself a member, nor do I now consider myself a member.

However, even if I had been allied to your Association in the interval, I say boldly, and without the slightest fear of an honest contradiction, that there is no act of mine which, after careful investigation, will be found in violation of the "code of ethics."

I do not offer this as an explanation or apology. Being familiar as I am with the men and motives prompting the charge, I will not deign an explanation. If either you gentlemen of the committee or the Association has any further curiosity as to the item in the newspaper, you may gratify the same by calling at the office of the paper in which the item appeared.

Trusting to the courtesy of the Board of Censors to spare me the necessity of taking other steps to prevent a repetition of this annoyance, I have the honor to be,

Very respectfully,

JAMES KENNEDY.

At this time threats of personal violence to me were made by the beligerent element of this chaste body. While the ire of the President and Board of Censors was at fever heat, I was interviewed in my office by the *Daily Express*, from whose columns the following is clipped:

"Dr. James Kennedy, at one time Secretary of the West Texas Medical Assocition, was seen yesterday in regard to rumors of the withdrawal of a number of the physicians from that Association on account of the resolution recently adopted suspending members who fail to attend three consecutive meetings. Dr. Kennedy readily accommodated the reporter. As he proceeded with his statement of affairs as he saw them, he became eloquent and enthusiastic at times, as though he had a number of the men he arraigns before him and was talking for their special benefit. Dr. Kennedy replied:

" 'Well, I think it is the last faint echo of the organization's death knell, which has been sounding for many months past. I am credibly informed that already a number of resignations have been handed in and a large number of members have signified their intention of resigning. A careful inquiry into the history of this society reveals the fact that very little has been accomplished the last few years towards unifying the profession or advancing the science of medicine towards that goal of perfection which it is the earnest hope of every true disciple of our noble calling that we will eventually attain. All honor to the founders of this now expiring body, for their zeal and energy as displayed in their efforts to advance the profession of medicine and surgery, and deep should be the regret of every one of us who has contributed our mite towards making the West Texas Medical Association the truly scientific and instructive organization, which it was the design of its founders it should be; to see its life blood sapped away by contention, jealousy and strife—to reflect that of the structure whose very foundation was built of love for science and a desire for mutual advancement and success, whose corner stone was "Do unto others as you would be done by," and whose pass word was "harmony," nothing should be left but bare and crumbling walls whose principal function now is to re-echo the calumnies that are cast upon those who absent themselves because they find such company and pastime uncongenial.

" 'Not even those who have grown gray in the harness and whose names are a synonym for ability, integrity and skill—who are honored, loved and revered by the people, escape slander and abuse. As to the younger men, if they fail to accept the *ipse dixit* or to pay tribute to the infallibility and profundity of the handful of egotists who have usurped the control of its affairs, they simply are not in it. If a young physician ventures to express an opinion, no matter how well founded on fact it may be, unless it be in accord with the preconceived notions of those who happen to be a few years his senior, he is summarily squelched—sat down upon as it were. This seems to be considered a just recompense for his timerity.

" 'The war cry of the "regulators" in this body is "medical ethics." The "regulators" are of course self-constituted—they are duly qualified doctors of medicine whom the public, it seems, has never sufficiently appreciated to keep them actively employed in practice—hence they have an abundance of time at their disposal and consume it in prating medical ethics and in finding fault with their busy brethren. They consider it a heinous offense to allow one's name to appear in print in connection with the report of any accident or operation, whether it be with or without the knowledge of the physician.

" 'The "regulators" emphatically state that such conduct (allowing one's name to be reported in connection with an accident case as a matter of news) is unprofessional, inasmuch as it is considered advertising, which is prohibited by the "code;" yet a glance at our daily papers, both English and German, discloses the fact that spaces have been taken by our great sticklers on ethics for the display of their cards, and on those pretty little silken banners which are hung up in the rooms of our hotels for the information of guests as to rules and regulations, etc., will also be found the advertisement of the present executive of the Association. Such beautiful consistency certainly merits admiration.

" 'But enough of this. Let the requiem be sung o'er this defunct body (the West Texas Medical Association) and from its ashes let there arise an organization whose code of ethics shall be the golden rule, whose aim shall be the advancement of the science of medicine and surgery, whose requirements for membership shall be scientific and practical worth. Let there be no regulators, but let every man regulate himself as becomes a man, a gentleman and a doctor. To such a society personalities, petty jealousies and dissension will be strangers. An organization founded upon this basis cannot fail to be productive of good to both the profession and the public.' "

After my distinguished brethren had devoured this choice literary morsel, they flew into a fury of passion, held a meeting, and expelled both myself and Dr. Caffery. Two or three cool-headed and fair-minded gentlemen who happened to be present remonstrated at the gross injustice of the contemplated act and pointed out the irregularity of such precipitate action in the matter. They called attention to the fact that no proofs sustaining the charge of "advertising" had been adduced. But the "wrathful seven" would not be appeased. Expulsion had been determined on beforehand, and expel us they must, and did, in direct violation of the constitution of the Association.

The article providing for the expulsion of members states that

"Any member may be expelled for immoral and unprofessional conduct by a vote of two-thirds of the members of the Association, provided that he receives notice in writing from the Secretary of the charges and specifications, and by whom preferred, at least thirty days before trial."

I will here state that I never received any communication from the Secretary whatever, and no notice of trial was ever given, nor was any trial ever held. And further, that there was not two-thirds of the members of the Association present, nor even one-tenth of its membership.

Supplementary to the above remarks I will quote the following:

SAN ANTONIO, TEXAS, April 16th, 1892.

*To Whom It May Concern:*

This is to certify that I caused to be inserted in the *Daily Express*, January 26th, a notice of an operation upon my daughter performed by Drs. James Kennedy and Russell Caffery, and that said notice was inserted without their knowledge or consent.

WM. ———.

Sworn to and subscribed before me this 16th day of April, A. D., 1892.
HENRY LAAGER,
[Seal.] Notary Public, Bexar County, Texas.

SAN ANTONIO, TEXAS, April 23rd, 1892.

*To the Texas State Medical Association:*

GENTLEMEN: We the undersigned physicians, practicing in the city of San Antonio, submit the following statement for the information of your Judicial Council, relative to the expulsion of Drs. James Kennedy and Russell Caffery from the West Texas Medical Association.

We were not present at the session when these gentlemen were expelled, but judging from such information as we have received concerning the matter, we do not believe that they were given a fair trial, or that the Association proceeded in the manner prescribed by its constitution.

H. J. TROLINGER,
EDWARD BENNETT,
H. D. BARNITZ,
RUDOLPH MENGER.

[Telegram.]

BOERNE, TEXAS, April 24.

*To James Kennedy, S. A.:*

I believe you were expelled in violation of by-laws.

DR. HERFF.

SAN ANTONIO, TEXAS, April 23, 1892.

*To the Texas State Medical Association:*

GENTLEMEN—We desire to state that we were present at the session of the West Texas Medical Association held on the 25th day of February, 1892, when Dr. James Kennedy and Dr. Russell Caffery were expelled from the society.

We also beg to state that there were but a few of the members present, and not a sufficient number to act on the expulsion of a member in accordance with the constitution of the Association.

Charges of advertising were preferred by the Board of Censors against Drs. James Kennedy and Russell Caffery, but were not sustained by any evidence; nevertheless, they were expelled.

It is our opinion that the trial was a farce, and the expulsions were unjust and unconstitutional.

EDWARD CROSS, M. D.,
F. E. YOUNG, M. D.

Sworn to and subscribed before me by Edward Cross, M. D., and F. E. Young, M. D., on this the 23d day of April, 1892.

[Seal] JAS. L. BURGESS,
Notary Public, Bexar county, Texas.

These are the facts in the case relative to my expulsion from the West Texas Medical Association.

The case was brought before the State Association by the West Texas Medical Association, for the avowed purpose of having me expelled from that body. Both the President of the West Texas Medical Association and the Board of Censors boasted that they would have me expelled from the State Association

With the facts before the Judicial Council as they are herein briefly recorded (and it seems to be a pretty clear "case," notwithstanding the open letter in the *Texas Health Journal* says there was "no case" before the Judicial Council, as the matter was forwarded to them for their "information" only), what else could they do, honorable men as they were, but to bring in a verdict of "not guilty," which they did in the following words:

REPORT OF JUDICIAL COUNCIL, TEXAS STATE MEDICAL ASSOCIATION.

TYLER, TEXAS, April 26, 1892.

In the matter of the expulsion of Dr. James Kennedy from the West Texas Medical Association, it was resolved that the papers submitted disclose irregularities and non-conformities to their by-laws and constitution of such a gross character that we cannot approve them without doing injustice to Dr. Kennedy; therefore we order that they be returned to the West Texas Medical Association for correction and legal adjudication at home before appealing to the T. S. M. A., and that Dr. Kennedy's proper recourse is, after due trial by the local Association, to this Association.

J. C. LOGGINS, M. D., Chairman.

The above is a correct transcript of the report of the Judicial Council upon the subject mentioned.

H. A. WEST. Sec. T. S. M. A.

Before closing this article, I beg to call your attention, Mr. Editor, and the attention of all who have followed the thread of this controversy, to the "purport of the following article which appeared (also) in a daily paper," Dr. Kingsley being the President and Dr. Harmer Vice-President of the society which construed the "purport" of the other newspaper paragraph to mean "a gross violation of the Code of Ethics." I am not advised as to whether or not the Board of Censors wrote these gentleman a "courteous letter" and demanded an explanation. It seems to be a matter of much moment whose ox is gored:

"IN A CRITICAL CONDITION.

"Mrs. W. R. Potter, residing at 292 East Commerce street, is lying at the point of death, and her recovery is doubtful. For some weeks she has been suffering with a tumor. Yesterday afternoon Drs. Kingsley and Harmer were sent for, and performed an operation, relieving her of the terrible agony which she had been having. Another surgical operation will be performed this afternoon, and it is thought that a chance to save her life is probable."—*San Antonio paper.*

In conclusion, I wish to remark that the uncalled for attack on Dr. West in the "official open letter" of the West Texas Medical Association, and their defiance of the authority of the State Association as expressed therein, which bears the autographs of the Secretary, the seal and the President (which triumvirate I am inclined to believe is all that now remains of this famous set of wranglers), is quite in keeping with the spirit that has always dominated in this, it is to be hoped, now extinct Association. Dr. West's well known high personal character places him beyond the range of his peurile assailants and their contemptible sallies, and needs no defense at my hands.

The portion of the record which they charge was abstracted, was my own personal property, and was returned to me at my own request, by the Judicial Council.

Some idea of the amount of dissatisfaction existing in the ranks of the West Texas Medical Association may be found in the resignation of the following members in the past year:

Drs. Bennett, Trollinger, Menger, Watts, Sommerville, Shropshire, Cross.

JAMES KENNEDY, M. D.

[P. S.—August 10th, the newspapers announce the withdrawal of Dr. C. E. R. King also, one of the Censors.—ED.]

